# An APC:WNT Counter-Current-Like Mechanism Regulates Cell Division Along the Human Colonic Crypt Axis: A Mechanism That Explains How *APC* Mutations Induce Proliferative Abnormalities That Drive Colon Cancer Development

**DOI:** 10.3389/fonc.2013.00244

**Published:** 2013-11-07

**Authors:** Bruce M. Boman, Jeremy Z. Fields

**Affiliations:** ^1^Center for Translational Cancer Research, Helen F. Graham Cancer Center and Research Institute, University of Delaware, Newark, DE, USA; ^2^Kimmel Cancer Center, Thomas Jefferson University, Philadelphia, PA, USA; ^3^CATX, Inc., Gladwyne, PA, USA

**Keywords:** adenomatous polyposis coli, WNT signaling, survivin, colon cancer stem cells, crypt fission, crypt cycle, adenoma morphogenesis, colonic stem cells

## Abstract

APC normally down-regulates WNT signaling in human colon, and *APC* mutations cause proliferative abnormalities in premalignant crypts leading to colon cancer, but the mechanisms are unclear at the level of spatial and functional organization of the crypt. Accordingly, we postulated a counter-current-like mechanism based on gradients of factors (APC;WNT) that regulate colonocyte proliferation along the crypt axis. During crypt renewal, stem cells (SCs) at the crypt bottom generate non-SC daughter cells that proliferate and differentiate while migrating upwards. The APC concentration is low at the crypt bottom and high at the top (where differentiated cells reside). WNT signaling, in contrast, is high at the bottom (where SCs reside) and low at the top. Given that WNT and APC gradients are counter to one another, we hypothesized that a counter-current-like mechanism exists. Since both APC and WNT signaling components (e.g., survivin) are required for mitosis, this mechanism establishes a zone in the lower crypt where conditions are optimal for maximal cell division and mitosis orientation (symmetric versus asymmetric). *APC* haploinsufficiency diminishes the APC gradient, shifts the proliferative zone upwards, and increases symmetric division, which causes SC overpopulation. In homozygote mutant crypts, these changes are exacerbated. Thus, *APC*-mutation-induced changes in the counter-current-like mechanism cause expansion of proliferative populations (SCs, rapidly proliferating cells) during tumorigenesis. We propose this mechanism also drives crypt fission, functions in the crypt cycle, and underlies adenoma development. Novel chemoprevention approaches designed to normalize the two gradients and readjust the proliferative zone downwards, might thwart progression of these premalignant changes.

## Introduction

It is well-known that APC down-regulates WNT signaling in normal human colon and that *APC* mutation impairs this down-regulation and contributes to the development of premalignant crypts, which leads to colon cancer [reviewed in (1, 2)]. However, the mechanisms are not well understood at the level of the spatial and functional organization of the colonic crypt. Therefore, we created a counter-current-like model that considers gradients of factors (APC; WNT) along the crypt axis that spatially and temporally regulate colonocyte proliferation and differentiation along this axis. To understand this problem and our proposed solution requires an understanding of the normal colonic crypt.

To better understand the role of APC, crypt renewal, and colonic stem cells (SCs) in maintaining normal form and function of the colon, we will first discuss the organization and function of normal colonic epithelium. This discussion is important because colonic SCs bequeath molecular information to their non-SC progeny that determines the structure and function of normal colonic epithelium. With that as a foundation, we can then begin to see how changes in populations of SCs can contribute, during colon tumor development, to altered tissue structure and altered tissue function. Although there has been much research on the structure and the function of rodent small intestine, which has increased our understanding of the biology of GI SCs, here we will emphasize knowledge obtained from human colonic SCs, human colonic epithelium, and human colonic cancers. If the reader wishes information in this field as it pertains to SCs in rodent tumorigenesis, several excellent reviews are available (3–5).

## Histologic and Proliferative Characteristics of Normal Human Crypts That Contain Wild-Type *APC*

Anatomically, colonic epithelium in humans is made of regular, pit-like structures called crypts, each containing two to three thousand cells (6, 7). The epithelium of the colon has very high turnover – it is replaced every 5 days through crypt renewal (6). Because the human colon contains ∼10^11^ cells (8) nearly 10 trillion colonocytes are generated per year. Remarkably, it is the colonic SC that underlies the generation of this large number of cells during an individual’s lifetime while, at any given time, maintains the number of crypt cells constant and crypt dynamics at steady state.

### The role of normal SCs in normal human crypt structure and function

The bottom of the crypt is where most colonic SCs reside. They are generally quiescent but do generate rapidly proliferating cells (transit-amplifying cells) that are simultaneously differentiating and proliferating (i.e., they are maturing) as they migrate upwards along the crypt axis. As they migrate, they are maturing along the various cell lineages such as absorptive (columnar) goblet (mucin-producing), and other cell types (9–12). Maturing cells, in turn, generate fully mature cells. These terminally differentiated cells continue migrating upwards, become apoptotic, and are eventually sloughed off, at the crypt top, into the colonic lumen.

Several mechanisms are in place to maintain the crypt size and the colonocyte population size constant. In dividing, the SC population at the bottom of the crypt undergoes self-renewal and, at the same time, generates the population of transit-amplifying cells (13). Because colonic SCs are long-lived, they are essential for crypt self-renewal over the lifespan of each individual. Extrapolations of findings from biologic studies in rodents suggests that SCs in a human colonic crypt are a small proportion (∼1%) of all cells in that crypt (14). This estimate is in accord with recent immunostaining experiments in human colonic crypts for the SC markers (15, 16). Nevertheless, these rare SCs drive crypt renewal and are key to crypt homeostasis and viability (17, 18).

### Dynamics of normal crypt cell populations

The dynamics of human colonic crypts are complex [reviewed in (19)]. (i) Crypts contain many cell types. (ii) Most crypt cells have neither a static location nor a static phenotype. As most crypt cell types migrate toward the crypt top, they proliferate and differentiate simultaneously (i.e., they undergo maturation). Eventually they become fully mature, no longer proliferate, become terminally differentiated, and, after apoptosis, are extruded at the crypt top. (iii) Not surprisingly, given the above, cell phenotypes change as colonocytes migrate and mature upwards along the crypt axis and various phenotypic markers show gradient-like distributions.

A few cells of the colonic crypt, the SCs, are different. (i) They don’t migrate upwards, remaining, instead, near the crypt bottom. (ii) They are multipotent. Human and rodent studies show that colonic SCs generate several lineages (endocrine cells, absorptive cells, goblet cells). Via tissue renewal, SCs replenish not only their own population, but also, all crypt cell types. (iii) SCs are extremely long-lived. Since crypts are closed systems, crypt cells must be generated by SCs that are already residing in the crypt. Therefore, both the number of cells in the normal crypt and the division of SCs require strict physiological regulation.

### Studying the generation of rapidly proliferating non-SCs by SCs

Because of numerous obstacles to the study of SCs in humans, initial studies focused on the functional properties of these cells. One of the earliest ways used to study SCs and to determine their anatomic location was pulse-labeling of DNA of rapidly proliferating cells – daughter cells that are produced by SCs. Uptake of bromodeoxyuridine (BrdU) or [^3^H]thymidine by these daughter cells in human colonic crypts results in *in vivo* labeling of DNA-synthesizing S-phase cells (6, 20–22). When the fraction (proportion) of S-phase (labeled) cells is plotted against cell position (i.e., against cell level) along the crypt axis, from the crypt bottom to the crypt top, the result is a skewed bell-shaped curve termed the labeling index or LI. In normal colonic crypts, the curve for the LI is low at the crypt bottom (level 1) and top (∼ level 82) and maximizes at approximately level 15. Sequential LI profiles were used to track these labeled colonocytes, which showed that they migrate from bottom to top, where they are then extruded. These tracking results indicate that SCs must reside at the crypt bottom. These profiles also indicate that there is a small fraction of cells in S-phase at the bottommost crypt levels (6, 23), where SCs are located. This is also consistent with literature reporting that SCs are relatively quiescent (24–26).

### Identification, distribution, and mode of cell division of human colonic SCs

To study important questions such as: what regulates the distribution of SC in the human colonic crypt or what is their type of cell division, it has been necessary to find accurate markers for human colonic SCs. This effort has relied on showing that SC markers fulfill certain criteria – ones that differ somewhat from criteria for establishing SC markers in rodents because validating SC markers by lineage tracing cannot readily be done for human tissues for ethical reasons. Thus, validation in humans generally relies on demonstrating characteristics of self-renewal, tumor-initiating ability, long-term repopulating capability, and capacity for multi-lineage differentiation (27). Based on these criteria several reliable markers (e.g., CD44, CD133, CD166, Musashi 1) have been established for normal and malignant human colonic SCs (15, 28–31).

Our own work (16) led to the discovery that ALDH is a marker for human colonic SCs. We found that ALDH positive colonic cells exhibit the known SC properties of anatomic localization and tumor-initiating ability: (a) immunohistochemistry identified a small subpopulation of ALDH1+ cells (∼5%) localized to the bottom of normal crypts (where SC reside) and (b) the Aldefluor assay was used to isolate a subpopulation of malignant colonic cells that generates xenograft tumors (also showing the ability for self-renewal). As few as 25 ALDH+ cells generated tumors while as many as 10,000 ALDH− cells from colorectal cancers (CRCs) did not form xenograft tumors. It was also shown that ALDH+ cells possess the SC features of long-term repopulating ability and multi-lineage differentiation (16). This was done by showing that isolated ALDH+ cells have the ability: (1) to be serially passaged long-term as xenografts in mice (and in colonosphere cultures) with continued isolation of ALDH+ cells, and (2) to differentiate into all of the different cell lineages found in colonic tissues based on histologic evaluation of the tumor xenografts. Similar findings have been published by others (32–34). Taken together, this information is consistent with the conclusion that ALDH1 is a SC marker in the colon of humans.

We then determined staining indices to quantify the distribution of colonic SCs (16). Indices for ALDH+ cells and those marked by other SC markers (CD133, CD44) showed that there is a gradient in the number of SCs upwards along the crypt axis. This gradient is similar to the exponential decrease in stemness with distance from the crypt bottom that we previously reported (23). The reason that this gradient exists is explainable in two ways. (i) In the first explanation, there are, upward along the crypt axis, decreases in the fraction of cells that are SCs. In this view, being a SC is *all-or-none*, and SCs can divide asymmetrically or symmetrically. The division of SCs can occur asymmetrically to produce one SC and one non-SC, symmetrically to produce two identical SCs, or symmetrically to produce two non-SC (35, 36). In theory, division of SCs must, on average, be asymmetric in order to maintain the SC number constant (37). That is, they must produce an average of one SC and one non-SC over all crypt cell divisions. Otherwise, the crypt cell population size will change, as it does in colon tumorigenesis, where SC overpopulation occurs (16).

(ii) In the second explanation, gradual decreases occur, upwards along the crypt axis, in the degree of stemness of each maturing cell. In fact, a radiobiology study (17) suggested that stemness is not all-or-none; rather, stemness is lost gradually. Hence it was postulated (38) that in early generations SCs gradually lose the capacity to function as SCs, and, eventually, all SC potential is lost. Other findings (39, 40) indicate that rodent intestinal SCs generate progenitor cells that have some SC-like properties and that become committed to differentiating along a particular cell lineage. This concept, that there are “intermediate degrees of stemness” is consonant with many of the latest rodent models of small intestinal SC with active SCs and ones that are recruitable, and where the “probability of stemness” represents a gradual change rather than a binary change (24, 41–43). That these progenitor cells seem to have intermediate degrees of stemness supports the idea that cells undergo gradual decreases in their degree of stemness as they migrate along the crypt axis and mature (23).

## Proliferative Changes in *APC* Mutant Crypts during Human Colon Tumorigenesis

The mechanisms by which APC mutations lead to CRC initiation have not been fully elucidated. For instance, it is not clear how a germline *APC* mutation can initiate intestinal tumors as it clearly does in *Apc^Min/^*^+^ mice and familial adenomatous polyposis (FAP) patients. Most sporadic CRC cases as well are initiated by *APC* mutations – such mutations are observed in ∼80% of cases of sporadic CRC (44, 45). Inactivation of the second *APC* allele happens during intestinal adenoma and carcinoma development in both *Apc^Min^* mice and FAP patients (46). This “second hit” typically results in the total absence of wt-APC protein (47, 48). However, in the case of homozygous mutant *APC*, the truncated APC protein usually contains some residual functions (discussed below) (48, 49).

Histopathologic studies on *APC* mutant tissues from FAP patients have been done to investigate how *APC* mutations might lead to development of CRC. An early finding was that proliferative mechanisms in the colonic crypt become dysregulated. The proliferative alterations were first shown several decades ago using pulse-labeling with BrdU or [^3^H]thymidine and plotting LI (labeling index) curves (20–22, 50). For normal-appearing FAP crypts, LI curves are shifted toward the crypt middle, maximizing at about level 20 (in normal colon, the maximum is at level 15) (50). This proliferative shift in normal-appearing (not yet dysplastic) FAP crypts is the earliest-known tissue alteration resulting from a germline mutation in the *APC* gene. Notably, crypts (e.g., FAP crypts) exhibiting this proliferative abnormality don’t show any microscopically visible changes in histology. Crypts begin to show abnormalities in histology only when they become dysplastic, i.e., during the formation, later, of premalignant adenomas, which have a second hit at the *APC* locus. For adenomatous crypts from FAP patients, LI curves are shifted even further up the crypt, toward the top (51, 52).

It is possible that the observed shift in the distribution of labeled cells reported in these studies might, in theory, have been caused as a result of variation in the length of the crypt (53–55). However, more comprehensive studies on humans have substantiated that this is not the case. In these studies (56, 57), fresh colonic biopsies from unaffected controls and FAP patients were pulse-labeled *ex vivo* with [^3^H]Thymidine. Moreover, the distribution of labeled cells was not determined based on “crypt level” (which could vary with crypt length) but on the “proportions” of cells along the crypt axis from bottom to top (which would not vary with crypt length). Zhang et al. (58) used a different approach to map the distribution of proliferating cells – namely using quantitative immunohistochemical (IHC) mapping of Ki67-labeled cells and by plots of staining indices. These mapping results showed that in FAP crypts the population of Ki67+ cells extended upward into the crypt middle as compared to distribution of Ki67+ cells in normal crypts where Ki67+ cells were restricted to the bottom-third. Similar results were found by Mills et al. (59). In adenomas from FAP patients, the shift was even more pronounced; cells staining for Ki67 were mostly found at the top of the crypt or on the luminal surface of the adenomatous epithelium. Thus, results from three independent approaches, quantitative IHC crypt mapping (58, 59), pulse-labeling of crypts *in vivo* (20–22, 50–52), and pulse-labeling of crypts *ex vivo* (56, 57), all provided support for the existence of an upward shift of the proliferative zone in normal-appearing and adenomatous crypts in FAP patients.

Other studies have investigated mitotic cells (rather than [^3^H]thymidine-labeled or BrdU-labeled cells) in FAP colonic crypts (60, 61). But mitotic indices have not been reported probably because scoring mitoses is difficult due to the small number (<0.5%) of mitotic figures per crypt. Wasan et al. (60) did report, using crypt microdissection, on the highest crypt level at which a mitotic figure was observed and found a modestly higher level in FAP patients than in normal patients. However, the difference was not significant, possibly because he was studying only a small series of FAP patients (*n* = 15). Mills et al. (61) studied a larger series (*n* = 29) using the same technique, crypt microdissection. They found a marked and significant (*p* < 0.0001) increase in the number of mitoses per crypt in FAP crypts (14.2) vs. control crypts (5.6). More recently, we (58) used quantitative IHC mapping of phospho-H3+ cells to measure the distribution of mitotic cells. This approach showed that in FAP crypts the population of mitotic cells extended upward into the crypt middle as compared to normal crypts in which phospho-H3+ cells were located in the bottom-third. In adenomas from FAP patients, the staining index for phospho-H3+ cells revealed that the shift of mitotic cells was even more pronounced for adenomatous crypts. Thus, these data on mitotic cells are consistent with the proliferative shift (based on LI) observed in FAP.

It was unclear, however, how *APC* mutations generate these earliest-known tissue events during colonic neoplasia development, that is, an upward shift along the crypt axis of the proliferative zone (as indicated by shifts in the LI curve). To investigate mechanisms as to how the proliferative abnormality occurs, we turned to mathematical modeling. Our modeling results (37, 62, 63) clearly demonstrated that only increases in crypt SC number, not alterations in apoptosis, differentiation or cell cycle proliferation of non-SC populations, could accurately simulate the LI shift in FAP crypts. This led us to postulate that the missing link between an *APC* mutation and the LI shift in the initiation of CRC in FAP trait carriers is the overpopulation of crypt SCs.

Biological studies (63, 64) that we did to follow up on our modeling study (37, 62) provided data to support this SC overpopulation mechanism, as have other studies. For example, using methylation pattern diversity, Kim et al. (65) found enhanced SC survival in FAP, which is consistent with SC overpopulation in CRC development. In another study (66), the orphan G protein-coupled receptor GPR49 (LGR5) was found to be overexpressed in primary human colon tumors and LGR5 was then found, in rodent studies (67), to be a SC marker. These research findings led us to determine that ALDH1 is a marker for human colonic SC and allowed us to demonstrate that SC overpopulation occurs due to an *APC* mutation during CRC development (16). Using ALDH1 also allowed us to track SC overpopulation in *APC* mutant tissues during the stepwise progression to CRC development in FAP patient tissues.

Molecular studies have also been done to elucidate APC-based mechanisms that contribute to CRC development [reviewed in (1, 2)]. In normal tissues, the APC protein controls WNT signaling by binding to the β-catenin protein in the cytoplasm, which in turn leads to β-catenin degradation. If *APC* is deleted or mutant, the degradation rate of cytoplasmic β-catenin is diminished. In a small proportion of CRC cases, ones that lack *APC* mutations, β-catenin (*CTNNB1*) mutations are found. Both *CTNNB1* and *APC* mutations activate Tcf4-mediated transcription. The increased levels of cytoplasmic β-catenin lead to increased binding to and activation of Tcf4 (Tcf/Lef) transcription factors, factors that regulate target protein expression and, in turn, cell proliferation and differentiation. For example, Korinek et al. (68) showed, in rodents lacking Tcf4, that epithelial SC compartments become depleted in small intestinal crypts. Other studies (69) showed that Apc modulates embryonic SC differentiation by controlling the dosage of beta-catenin signaling. Moreover, LGR5 that is overexpressed in human CRCs (66) is a Tcf4 target gene (70) and LGR5 was then used to identify crypt SCs as the cells-of-origin of intestinal cancer (71). Taken together, these findings show that *APC* mutations and activation of WNT signaling pathways are crucial to the development of CRC.

## APC and WNT Gradients

**Figure 1:**
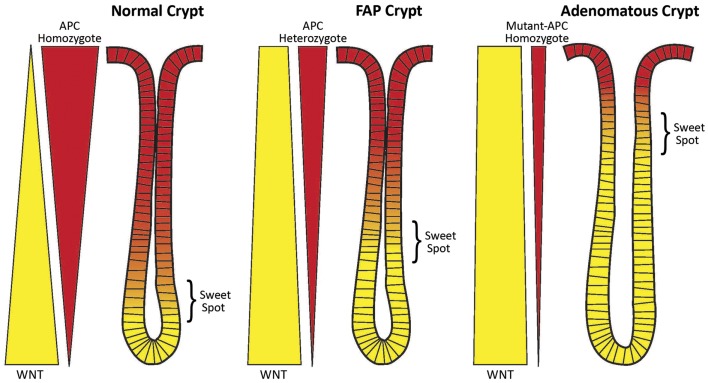
**Schematic of the counter-current-like mechanism**. The “sweet spot” marks the crypt region where levels of APC and WNT signaling are balanced and optimal conditions exist for mitosis and maximal cell proliferation. Both APC and WNT signaling components (e.g., survivin) are essential for mitosis. Left Panel: In normal crypts (wt-*APC* homozygote), the gradients of WNT signaling (yellow wedge) and APC signaling (red wedge) are balanced and the “sweet spot” is in the lower crypt. Middle Panel: In FAP crypts (wt-*APC* heterozygote) the situation has changed. Patients with FAP have a germline heterozygous *APC* mutation and thus a 50% reduction in *APC* gene dosage. Therefore, there is 50% less APC protein expressed (as indicated by the narrower red wedge), and less suppression of WNT expression and WNT signaling (as indicated by the wider yellow wedge). The balance point, that is the “sweet spot,” has been shifted to a higher crypt level. Right Panel: In adenomatous crypts (mutant-APC homozygote), the changes in WNT expression (still wider yellow wedge) and APC expression (still narrower red wedge) are exacerbated due to a sporadic APC mutation in the second APC allele (the second hit). In mutant-APC homozygote cases truncated APC protein can retain some residual function. Here the “sweet spot” is shifted even further up the crypt. A consequence of these changes is an increase in the number of immature cells (including SCs) in the crypt. The SC overpopulation is thought to drive colon tumorigenesis.

How crypt SC overpopulation is caused by *APC* mutations remains unclear. An explanation we are presenting here is that, in the normal crypt, APC-induced down-regulation of WNT signaling establishes an APC:WNT gradient and dysregulation of this gradient in tissues containing *APC* mutations is key. In normal crypt renewal, daughter cells produced by SCs at the bottom of the crypt proliferate while they migrate upwards. Because APC protein produced in crypt cells increases as the cells migrate upwards (58, 72–80), APC concentrations are low at the bottom of the crypt (where SCs reside) and high at the top of the crypt (where differentiated cells are). In contrast, WNT signaling is greater at the bottom of the crypt, occurring through a complex network consisting of different WNT ligand and receptor signaling components (81, 82). Activation of WNT signaling in the crypt bottom was shown by studies demonstrating accumulation of nuclear TCF4 in the crypt proliferative compartment (70, 83–85). There are several lines of evidence demonstrating that continual stimulation of the WNT pathway in the crypt bottom is essential for maintenance of intestinal SCs, normal proliferation of transit-amplifying cells, enterocyte maturation, and crypt homeostasis (86, 87). WNT gradients are high at the crypt bottom and low at the crypt top (inverse to the APC gradient). Given the existence of these inverse gradients and the dynamics of their interactions, we construed that there is a counter-current-like mechanism in the normal crypt and that this mechanism likely regulates changes in cellular phenotype associated with colonocyte maturation along the crypt axis (Figure 1).

## Counter-Current-Like Mechanisms

Counter-current mechanisms are found extensively in nature. Typically, the incoming and outgoing components flow in opposite directions to each other and interact to retain a high concentration of a substance at one point in the system. In the colonic crypt, one component is the Wnt gradient and the other is the APC gradient. These gradients are not only “counter” to each other, but also APC and Wnt are both necessary for proliferation (but neither is sufficient). As discussed below, APC and Wnt components are known to interact during mitosis and, in our model, this interaction maintains cell division at a high level in a specific area in the lower region of the normal crypt (peak at approximately crypt level 15).

This raises the question as to what maintains the APC and Wnt gradients in the normal colonic crypt. The inverse pattern is consistent with their being feedback and/or feed-forward regulation, which is a key to many counter-current mechanisms. For example, Wnt signaling is activated at the crypt bottom by a complex network of various Wnt ligands and receptors. The Wnt and APC gradients can even affect each other. Indeed, it is well-known that APC down-regulates Wnt signaling.

### Regulation of APC expression

Of course the question arises: What factors regulate *APC* gene expression and is WNT signaling one of those factors? One factor appears to be cell proliferation. For example, Umar et al. (75) found that epithelial proliferation induces APC expression and full-length APC protein increases during rodent intestinal epithelial hyper-proliferation. Fagman et al. (88) showed a similar effect. Their study showed that nuclear accumulation of full-length and truncated APC protein in colon carcinoma cell lines depends on proliferation. Another factor is that regulation of APC expression depends upon promoter methylation. Deng et al. (89) showed that methylation of CpG sites around a CCAAT box in APC’s promoter region inhibits APC’s gene expression by changing chromatin conformation and interfering with the binding of transcription factor CBF (CCAAT binding factor) to the CCAAT box. Studies on various cancers provide further support for the idea that expression of APC is affected by promoter methylation. Indeed, *APC* promoter hyper-methylation has been found to occur in a variety of human cancers including breast (44%) and lung (53%) and other cancers (90). This promoter hyper-methylation leads to epigenetic inactivation of the *APC* gene. Some transcription factors such as p53, USF1, USF2, and GC-box binding protein Sp3 have also been shown to regulate *APC* gene expression (91–95). While it has not been shown (to our knowledge) that Wnt signaling directly regulates APC expression (APC is not a known TCF4 target gene), WNT signaling does have a role in chromatin remodeling via beta-catenin’s interaction with chromatin remodeling complexes that can affect gene transcription (96). This role fits with the observation that chromatin is more condensed in cells at the crypt base, the region where WNT has highest activity (97). Thus, it is changes in chromatin conformation (open or closed) along the crypt axis that may modulate APC expression by affecting the ability of specific transcription factors to bind to *APC’s* promoter region.

## APC and WNT Signaling are Both Required for Mitosis

In particular, the existence of inverse APC and WNT gradients and their interactions begins to explain how crypt cell dynamics such as cell division are regulated. For instance, APC is a protein located at specific sites within mitotic cells and is essential for cell division [reviewed in (98, 99)]. Cell division also requires WNT signaling. In the WNT signaling pathway key down-stream signaling components include survivin, aurora B kinase (ABK), INCENP, and phospho-histone H3 (58, 100). ABK becomes activated when it forms a complex with the other three proteins. Like APC, ABK, Survivin, INCENP, and Borealin are also located at specific sites within mitotic cells and these proteins are necessary for cell division (101–103). We and others found that survivin is a TCF4 target gene (58, 63, 100, 104–106). Thus, APC itself down-regulates, via beta-catenin/TCF4, expression of survivin. This, in turn, modulates ABK activity, which contributes to the regulation of mitosis.

As both APC and WNT signaling are essential for mitosis, and as their gradients are inverse to one another, there has to be a zone along the crypt axis where the concentrations of APC and WNT pathway components are together optimal for maximal cell division – termed here a “sweet spot.” To better understand the underlying mechanisms of this phenomenon, we created a new model. It is a counter-current-like model that considers gradients of factors (APC; WNT) that regulate colonocyte proliferation along the crypt axis (Figure 1). The scientific basis of this model derives from the fact that both APC and WNT signaling are both required for mitosis, which we will now discuss.

### Role of APC in spindle orientation and microtubule function during mitosis

During mitosis, APC becomes localized to and acts at four subcellular sites: midbody, centrosomes, cortex, and kinetochores [reviewed in (98, 99)]. APC proteins have several mitosis-related functions. (a) APC acts at the plus ends of microtubules, which, in turn, interact with kinetochores. This increases kinetochore ability to attach to microtubules. APC also helps maintain mitotic fidelity – APC is needed for the normal function of the mitotic spindle checkpoint, which includes detection of transiently mis-aligned chromosomes. (b) At the cortex, APC regulates the stability of astral microtubules, and provides a cortical location for attachment of those microtubules. APC and other interacting cortical factors (which includes dynein) rotate the mitotic spindle into a defined orientation, and help orient the mitotic spindle. (c) In vertebrates, APC and EB1 (its binding partner) co-localize to the mother centriole, and this anchors a subset of microtubules. At this site, it is likely that APC has a role in centrosome re-orientation and directed migration. (d) APC also co-stains with tubulin at the midbody (which separates daughter cells) suggesting a role for APC in cytokinesis (107).

APC is associated, during mitosis, with kinetochores in metaphase, with the midbody during telophase and with polar microtubules in anaphase. During metaphase, APC interacts with EB1 in the alignment of chromosomes through plus-end capture and through attachment of microtubules to kinetochores (108, 109). This linkage seems to require EB1 and, notably, APC and EB1 localize specifically to the mother centriole. APC is also localized to the centrosome, and helps nucleate and anchor microtubules, which is required for establishment of the bipolar spindle.

### Role of WNT signaling in mitosis and mitotic spindle orientation

Survivin, ABK, INCENP, and Borealin are also involved in mitosis and function as a complex of chromosomal passenger proteins [chromosomal passenger complexes (CPCs)] localized to chromosomes in prophase (101–103). In metaphase, survivin targets this CPC to centromeres. The complex is stabilized by INCENP. Survivin binding to INCENP is promoted by Borealin. During metaphase, ABK is localized to the inner centromere. ABK regulates kinetochore-microtubule interactions and promotes proper chromosome bi-orientation by regulating and correcting kinetochore-microtubule attachments. In particular, ABK at the inner centromere inhibits formation of syntelic microtubule attachments, thus promoting monotelic attachments and appropriate bi-orientation on the mitotic spindle.

### APC, EB1, survivin, and ABK localize to similar sites during the different phases of mitosis

The cellular location of APC, EB1, survivin, and ABK during the different phases of mitosis has been described in several reviews (98, 99, 102).

In anaphase APC is located at the cortex and helps position the spindle. This depends on guiding cortical microtubules to specific cortical sites. This seems to require a microtubule plus-end protein complex, which includes APC, beta-catenin, EB1, and other proteins. APC is linked to microtubules by binding to EB1, and dynactin/dynein complexes are tethered to APC-associated EB1 at cortical attachment sites. Indirect links between APC and actin filaments seem to be mediated by beta-catenin and alpha-catenin, which provides, during mitosis, a functional link between microtubule and actin cytoskeletons. Survivin and ABK concentrate, during anaphase, at the spindle midzone and equatorial cortex in preparation for their roles in late mitotic events.

In telophase, APC co-stains with tubulin at the midbody (107). It is unknown how APC contributes to furrow induction during cytokinesis. But it may help guide cortical microtubules to the cortex, or control actin dynamics at the cortex. ABK and survivin also play critical roles in cytokinesis. During telophase, ABK and survivin are localized to the midbody. ABK seems to mediate cytokinesis by phosphorylating several proteins that localize to the cleavage furrow, which destabilizes intermediate filaments prior to cytokinesis (110, 111).

### Do APC, survivin, and ABK interact directly?

The fact that APC, EB1, survivin, and ABK localize to similar sites during the different phases of mitosis suggests that interactions occur between these proteins. Indeed, the APC binding protein EB1 provides a link between APC and ABK because EB1 and ABK co-localize to the central spindle in anaphase and to the midbody during cytokinesis. For instance, it was found that EB1 promotes ABK activity by blocking its inactivation by protein phosphatase 2a (112). Therefore, EB1 mediates microtubule dynamics in association with APC and also positively regulates ABK activity. In addition, formin mDia3, another APC binding protein, helps stabilize microtubule-kinetochore attachments and chromosome alignment in metaphase (113). This ability has been attributed to the binding of mDia3 to EB1, the other protein that interacts with APC. This provides another link to ABK because microtubule binding to kinetochores via mDia3 is regulated by ABK phosphorylation of mDia3 (114). Thus, during mitosis, there are both anatomical and functional links between APC, EB1, and ABK.

The evidence thus indicates that these key mitotic components need complex regulation during the different phases of mitosis and at different locations along the crypt axis. One aspect of this regulation, we and others found, involves APC itself. APC down-regulates expression of survivin via beta-catenin/TCF4, which, in turn, modulates ABK activity (58, 63, 100, 104–106). APC, which is a tumor suppressor gene, not only helps in mitosis but also promotes both differentiation and apoptosis in the colonic crypt. As noted above, APC in the crypt is distributed along a gradient, from essentially negligible at the crypt bottom to a maximum at the crypt top. The WNT gradient is inverse to the APC gradient. Since survivin is a down-stream component of WNT signaling, and survivin activates ABK, it is not surprising that survivin and ABK gradients are, like the WNT gradient, highest at the crypt bottom and lowest at the crypt top [(58, 100)].

That these gradients are inverse to one another might be seen as contradicting the fact that APC, ABK, and survivin are essential for appropriate progression of cells through mitosis. However, what this evidence really provides is insight into how cells undergo phenotypic transitions as they migrate and undergo maturation upwards along the crypt axis. In that view, there is, along the normal crypt axis, a region where APC, EB1, survivin, and ABK levels together generate the highest rates of proliferation and differentiation. In this “sweet spot,” transit-amplifying cells proliferate and differentiate – a concept that is supported by our mathematical modeling and biologic data (23, 58, 62, 63).

## Our Counter-Current-Like Mechanism is a Model That also Explains the Proliferative Changes during Colon Tumorigenesis

In heterozygous *APC* mutant crypts, such as normal-appearing colonic epithelium in FAP patients, there is half the wild-type *APC* gene dosage. But both APC alleles will still be regulated as in the normal crypt. However, one of the transcripts will be a mutant transcript and be translated into a mutant protein (50%) while the other will encode a wild-type protein (50%). Thus, the encoded wild-type protein levels should be reduced by about half at all points along the haplo-insufficient crypt axis and the gradient becomes diminished but retains its distribution. Because concentrations of wt APC are decreased, the optimal concentrations that generate the sweet spot occur further up the crypt (Figure 1). This upward shift in the position of the sweet spot parallels the shift in the labeling index in FAP crypts from the lower region to the middle crypt (see Proliferative Changes in APC Mutant Crypts During Human Colon Tumorigenesis, above).

In homozygous mutant *APC* crypts, such as are found in adenomas, these changes become exacerbated further. In this case, the APC gradient is not totally lost because the truncated APC protein usually contains some residual function (48, 49). Thus, optimal conditions corresponding to the sweet spot are found even further up the crypt. Indeed, the labeling index in adenomatous crypts shifts to the top of the crypt.

### Residual APC function is retained in cells with homozygous mutant *APC*

It is fascinating how APC mutation leads to retention of residual activity in the encoded mutant protein. In the situation where tumors are homozygous mutant for *APC*, the site of the “first hit” in the *APC* gene determines the type of the “second hit,” both in hereditary (FAP) and sporadic colorectal tumors (48, 115–118). This results in expression of truncated APC protein in most tissues with homozygous mutant *APC*. But, the truncated APC protein actually retains a microtubule binding domain (Armadillo repeats) and one to three intact β-catenin-binding amino acid repeats. This indicates that second hits at the *APC* locus occur that generate a “just-right” level of WNT/beta-catenin signaling that is optimal for tumorigenesis, with the combined hits (or “just-right” genotypes) resulting in only partial loss of APC functioning (49, 119, 120).

Therefore, in neoplastic crypts one has to look higher up the crypt to find the optimal APC concentration for cell division. This is also true for WNT because diminished APC leads to diminished down-regulation of WNT. Thus, it is only higher up the crypt where the APC concentration is high enough to diminish WNT levels to what normally was the optimal WNT concentration for promoting cell division. Shifting the proliferative zone upwards in neoplasia will theoretically increase symmetric SC division below the sweet spot, which will cause CSC overpopulation and promote colon tumorigenesis. Mutations at the second *APC* allele would exacerbate these changes. Thus, *APC*-mutation-induced changes in a counter-current-like mechanism will increase the number of proliferative cells (SCs, rapidly proliferating cells), contributing to colon cancer initiation and adenoma development.

Indeed, FAP crypts have increased mitoses (61), and a cardinal pathologic feature of colonic adenomas and carcinomas is increased numbers of mitotic figures and aneuploidy. One explanation for this increase is that the residual APC activity in combination with increased WNT signaling in neoplastic tissues increases the frequency of mitosis. If APC is necessary (but not sufficient) for mitosis, and if there is enough residual APC function in a tumor, one would expect more frequent mitoses (but not a greater rate of mitosis) when WNT signaling is also upregulated. In the setting where you have increased frequency of mitosis, you could also have changes in the fidelity of chromosome segregation. It is probably the dynamic interplay between APC and CPC proteins during mitosis that affects the fidelity of mitosis (e.g., the accurate segregation of chromosomes). Since the truncated APC protein is not fully functional, and leads to, via WNT, aberrant down-regulation of CPC protein expression, it is not surprising that *APC* mutations lead, in colonic tumors, to aberrantly oriented mitotic spindles and aneuploidy.

## Effects of Changes in Colonic APC and WNT Gradients in Neoplastic Crypts on Symmetric and Asymmetric Cell Division in the SC Niche

The counter-current-like mechanism may also play a role in regulating the symmetry of crypt cell divisions. The orientation of the mitotic spindle axis of colon cells appears to change upward along the normal crypt axis. In the crypt bottom, the mitotic spindle orientation lies perpendicular to the apical surface – an orientation that selectively occurs in the SC niche of human and rodent small and large intestine (121). This perpendicular alignment of mitotic spindles correlated with the pattern of retention of label-retaining DNA in the crypt base, a pattern that is consistent with asymmetric division of SCs. In the normal crypt middle (i.e., in the crypt column), where SCs are rarely found, Quyn et al. (121) showed that mitotic spindle alignments are mostly parallel to the apical surface, which is a pattern that is consistent with symmetric differentiated cell division. This mechanism involving change in the mitotic spindle orientation along the normal crypt axis could contribute to the maintenance of a constant number of SCs in the SC niche (16, 64).

In premalignant heterozygous mutant *Apc* tissue in rodents (*Apc*^Min/+^ intestine), both perpendicular spindle orientations and asymmetric DNA label retention were lost in the SC niche (but not in the crypt middle) (121). This pattern is consistent with a decrease in asymmetric and an increase in symmetric cell division in the SC niche in crypts with an *APC* mutation. Moreover, their murine small intestine and human colon data demonstrate that crypts with *APC* mutations show increased asymmetric cell division in the crypt middle (column). These data thus indicate that a shift in asymmetric cell division from the crypt bottom to the crypt column happens in parallel to the shift in the labeling index that was reported by others (20–22, 50, 56, 57). We also reported (58) that the subpopulation of cells expressing the mitotic proteins ABK and survivin shifts upward in FAP and *Apc*^Min/+^ crypts. The global effect on the crypt of an *APC* mutation thus is a delay in phenotypic transitions along the crypt axis, an increase in the number of SCs that divide symmetrically, and expansion of the SC population at the crypt bottom, which drives colon tumorigenesis.

## Studies on Rodent Intestine That Might Provide Insight into Mechanisms of SC Division in Human Colon

Theoretically, in normal tissue renewal, asymmetric cell division maintains the number of SCs constant (37, 122). An alternative concept is that SCs must, on average, have asymmetric divisions, even if each particular SC division is not always asymmetric (123). In neoplastic tissue, in contrast, it can be deduced that, in the development of SC overpopulation during human colon tumorigenesis, the rate of symmetric SC division must be increased (37). However, it is controversial whether, in the process of crypt renewal, SCs normally divide symmetrically or asymmetrically. Yatabe et al. (124) used methylation patterns to investigate this question for human colonic SCs. The patterns better supported a model of the crypt SC niche in which SCs were periodically replaced via symmetric SC division. And, using this methylation pattern diversity analysis, Kim et al. (65) found enhanced SC survival in FAP, which is consistent with SC overpopulation in CRC development. In contrast, a recent paper by Bu et al. (34) reports data showing that human colon cancer SCs can divide by either symmetric or asymmetric division.

There is now a vast literature emerging on mechanisms of SC division in rodent intestine that provide insight into mechanisms of SC division in human colonic crypts. As noted above, Lgr5 was identified to be a SC marker in mouse intestine (67). Using lineage-tracing models that were based on fate mapping, Snippert et al. (125) reported that rapidly cycling small bowel SCs in rodents (i.e., Lgr5+ cells), undergo symmetric SC division that follows a pattern of “neutral drift dynamics.” These findings support a stochastic mechanism in which symmetric SC division occurs in response to loss of a neighboring SC and, as expansion of the surviving clone continues, the crypt SC niche becomes increasingly monoclonal (126). Schepers et al. (127) studied SCs (Lgr5+ cells) in the base of the crypt looking for asymmetric segregation of chromosomes and asymmetric segregation of chromosomes was not observed at the crypt base since Lgr5+ intestinal SCs randomly segregated newly synthesized DNA strands.

Other studies, however, indicate that SCs divide asymmetrically. Asymmetric division of SCs in the SC niche supports a deterministic mechanism whereby a small number of SCs each generate a SC and a non-SC (a transit-amplifying cell). The non-SC daughter leaves the SC niche, and proliferates (promoting tissue renewal) whereas the SC daughter stays in the SC niche (35, 128). Asymmetric SC division is consistent with the “immortal strand hypothesis,” that is, the idea that during SC division, newly synthesized DNA strands segregate with the non-SC daughter to avoid mutations that are caused during DNA replication (129).

The “immortal strand” hypothesis has been tested using DNA-labeling methods to identify label-retaining cells (LRCs). Cells that retain DNA labels like BrdU or [^3^H]thymidine are thought to be SCs. Potten et al. (130) used double-labeling of cells in rodent small bowel using BrdU and [^3^H]thymidine (^3^HTdR). Template DNA strands in SCs were labeled with ^3^HTdR during tissue regeneration or development. Newly synthesized strands were labeled with BrdU, which established a way to follow how the two markers segregated after cell division. The authors found that the template strands (which were ^3^HTdR-labeled) were retained, but newly synthesized strands (which were BrdU-labeled) were lost following the second SC division. Studying cultured cells that cycle with asymmetric cell kinetics, Merok et al. (131) reported co-segregation of chromosomes containing immortal DNA strands, and that is also consistent with the immortal strand hypothesis. Moreover, as discussed above, Quyn et al. (121) observed that labeled DNA was asymmetrically retained in the SC niche of rodent intestinal crypts. The pattern of retention of label-retaining DNA correlated with the perpendicular alignment (alignment relative to the apical surface) of mitotic spindles in the crypt base.

In a study on rodent colon, Kim et al. (65) showed that a double-labeling method (BrdU and ^2^H_2_O) could be employed to identify and isolate nuclei from colonic epithelial LRCs. This let them measure proliferation rates of LRCs *in vivo* (*t*_1/2_ ≈ 140 days). Falconer et al. (132) used fluorescence *in situ* hybridization and unidirectional probes specific for centromeric and telomeric repeats. They found that one can identify parental DNA template strands in sister chromatids of rodent metaphase chromosomes. These findings showed that orientation of chromosomes is uniform; the 5′ end of the short arm is on the same strand as the “T-rich” major satellite repeats. This repetitive DNA orientation allows both analysis of mitotic segregation patterns and differential labeling of sister chromatids. The authors uncovered substantial non-random segregation of sister chromatids in a subpopulation of colonic crypt epithelial cells, which included cells outside the SC niche. This finding suggested that there exists in colonocytes a mechanism that controls how sister chromatids are allocated as intestinal SCs divide. Interestingly, DNA methylation is emerging as a mechanism that might regulate non-random template strand segregation suggesting that this aspect of SC division may be under dynamic control.

How can one reconcile these different findings regarding symmetric SC division and asymmetric SC division in the population of intestinal SCs during crypt renewal? One possible way is based on recent findings that, in the SC niche, different subpopulations of SCs exist. Using a variety of markers (Bmi1, Lgr5, and mTert), distinct intestinal SC populations were identified (133). Lgr5 labels SCs that rapidly cycle and are located in the crypt base, modulated by Wnt signaling, and sensitive to irradiation (71). Subsequently, using lineage-tracing experiments in adult rodents, Barker et al. (134) showed that cycling Lgr5+ cells are very long-term self-renewing cells in the intestine. Another study identified Lrig1 protein, the pan-ErbB negative regulator, as a specific intestinal SC marker which also functions as a tumor suppressor (135). In a different study, Montgomery et al. (136) identified a subpopulation of intestinal SCs that express telomerase reverse transcriptase (mTert), that cycle slowly, and that give rise to Lgr5+ cells. This study showed that although Lgr5+ intestinal SCs represent a different subpopulation, they can also have high telomerase activity. Bmi1 labels a different subpopulation of intestinal SCs. These SCs are quiescent, insensitive to Wnt signaling, resistant to high-dose radiation, and generate all the differentiated lineages in the crypt. Since Bmi1 and Lgr5 label two different populations of SCs and since Bmi1+ cells can generate Lgr5+ cells, Bmi1+ cells represent a reserve SC population that cause Lgr5+ cells to be dispensable (137). Thus, based on the above findings, the idea that SCs are quiescent has been challenged. Some studies suggest that there are slowly cycling SCs; other studies suggest that there are actively cycling SCs; yet other studies suggest that there are only relatively quickly cycling SCs (138–140). This evidence demonstrates that there may be different SC subpopulations in the intestinal SC niche and suggests that these different populations may even have different modes of division (asymmetric SC division vs. symmetric SC division).

Another possible explanation is provided in a recent editorial by Winton (141) in which he states “Interpretation of the growth dynamics of stem-cell-derived clones has previously demonstrated that symmetric fate choice is a common feature of intestinal SC self-renewal (126). However, asymmetric fate choices could be interspersed with symmetric ones and still be compatible with these models [e.g., if small numbers of SC per crypt are assumed; (125, 142)].” Another possible explanation is given by a recent study by Bellis et al. (143). These investigators found that Apc controls planar cell polarities that are central to gut homeostasis. By studying the SCs at the bottom of intestinal crypts, they discovered that spindle alignment and planar cell polarities form a functional unit that can generate daughter cell anisotropic movement away from niche-supporting cells. By proposing a mechanism involving anisotropic daughter cell movement rather than spindle re-orientation in SCs [per (121)], the Bellis model provides an alternate mechanism for the idea of neutral competition of SCs for niche-supporting cells, that is central to the concept of stochastic population asymmetry (128).

Alternatively, there are other studies that may reconcile the ambiguity as to whether SCs are rapidly cycling or quiescent. Buczacki et al. (24) found that quiescent intestinal cells in rodents are precursors that are committed to maturing into differentiated secretory cells of the Paneth and endocrine lineages. However, upon intestinal injury, they become capable of extensive proliferation and give rise to the other intestinal cell lineages. Thus, quiescent intestinal crypt cells represent a reserve population that can be recruited to a SC state. A study by Takeda et al. (43) also showed that there is inter-conversion between intestinal SC populations (Hopx+ slow cycling SCs and Lgr+ proliferating SCs) in distinct niches. Kobayashi et al. (144) reported that Lgr5+ colon cancer SCs interconvert with drug-resistant Lgr5− cells, which are capable of tumor initiation. Glauche et al. (145) reported that SC proliferation and quiescence were two sides of the same coin. They concluded that “hematopoietic SC organization was an adaptive, regulated process where the slow activation of quiescent cells and their possible return into quiescence after division are sufficient to explain the simultaneity of occurrence of self-renewal and differentiation.”

Clearly, a great deal of research has been done to study the various intestinal SC populations (e.g., Lgr5, Hopx, mTert, Bmi1, Lrig1, etc). However, the interacting dynamics and modes of division of these different intestinal SC types do not appear to be fully resolved. Taken together, we believe that these studies suggest that the intestinal SC is a cell that is in one of several phenotypic states that immature enterocytes can assume based on the dynamics within the colonic crypt. These dynamics may be relevant to those proposed in our counter-current-like mechanism proposed for human colonic crypts.

## Counter-Current-Like Mechanisms and Adenoma Development

**Figure 2:**
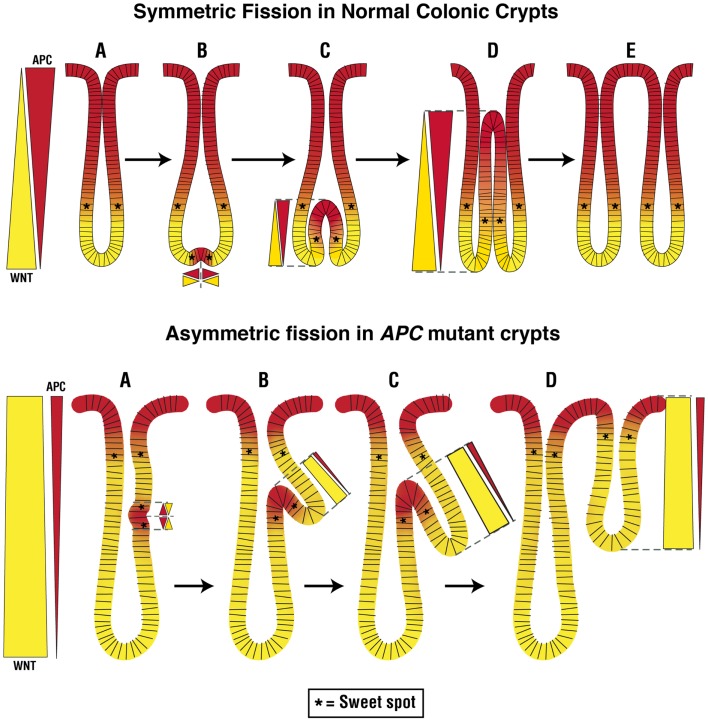
**Normal and abnormal crypt fission cycles**. The upper panel in the figure depicts the normal crypt cycle – a slow, continuous replication process involving growth (A), budding (A → B), bifurcation (B → D), and fission (D → E) phases. In the growth phase, crypts gradually grow in size until they transition to the budding/bifurcation phase. Fissioning then occurs in a symmetric manner through a budding mechanism that is triggered at the base of the crypt. Crypt bifurcation then longitudinally extends upward and crypt fission finally occurs to create two new, virtually identical, crypts. Our model predicts that at the place where budding first develops at the crypt bottom, cell division is enabled due to creation of a pair of new sweet spots. This is due to induction of APC expression at the crypt bottom that normally has a high background level of WNT signaling, which creates two new counter-current-like gradients of APC and WNT. The optimal conditions that support the high rate of proliferation in the new sweet spots provide the mechanism for a high rate of cell proliferation that expands the colonocyte population which drives the growth of the bifurcation upwards. The lower panel in the figure depicts the abnormal crypt cycle. In crypts with mutant APC, the rate of crypt fission is increased and fission often occurs asymmetrically. In this case, fissioning occurs because the budding/bifurcation starts somewhere along the crypt axis but not at the crypt bottom. Consequently, creation of two new counter-current like gradients of APC and WNT along the crypt column leads to generation of two new sweet spots which drive growth of the bifurcation upwards in an asymmetric fashion. The result of this abnormal crypt fission is two crypts of different sizes.

Adenoma morphogenesis is due, in large part, to abnormal crypt fission. In the normal adult rodent intestine and human colonic crypt, fission is responsible for regular replacement of crypts through the “crypt cycle” (146–150). The crypt cycle is a slow, continuous replication process involving three phases (growth, budding/bifurcation, and fission) (Figure 2, upper panel). In the growth phase, crypts gradually grow in size until the transition to the budding/bifurcation phase. The fissioning process then occurs in a symmetric manner through a budding mechanism that is triggered by a development of a bud (appearing as an indentation) at the base of the crypt. Crypt bifurcation then longitudinally grows and extends upward and crypt fission finally occurs to create two new virtually identical crypts (60, 151, 152). Two factors have been proposed to govern crypt fission: the size of the crypt and the size of the crypt SC population (147, 153). Since the crypt cycle produces expansion in the crypt population size, it is critical for epithelial homeostasis, as well as for repair of mucosal injury.

In adenomas that develop due to *APC* mutations, tissue disorganization is manifest as dysplasia, and premalignant tumor growth results from an increased rate of intestinal crypt fission (151, 154–156). For example, Wasan et al. (60) showed that both FAP patients and *Apc^Min/^*^+^ mice have increased rates of intestinal crypt fission in *APC* haplo-insufficient intestinal epithelia. In homozygote *APC* mutant epithelium, the rate of crypt fission is even greater (153). This identifies *APC* as one of the key factors in the regulation of crypt fission. Moreover, an increase in the crypt fission rate appears to account for the clonal and exponential expansion of mutant cell populations that drive tumor growth (157, 158). In normal-appearing and adenomatous intestinal tissues from FAP patients and *Apc^Min/^*^+^ mice, histologically aberrant crypt fissioning occurs. In this process, the budding/bifurcation process is asymmetrical, giving rise to crypt branching and non-identical crypts (60, 153).

So how might these changes in crypt fission relate to our counter-current-like model? In normal crypts, budding initiates the fissioning process at the bottom of the crypt. In our model (Figure 2), fissioning normally begins below the sweet spot at a point in the SC niche where WNT signaling is highest and APC is lowest. In this scenario, the APC/WNT gradients restrict the region where fissioning can be initiated to the crypt bottom, such that fissioning will proceed symmetrically.

In heterozygote and homozygote *APC* mutant crypts, asymmetric crypt fissioning appears to occur because fissioning starts anywhere along the crypt axis, not just at the crypt bottom (60, 159). In our model (Figure 2), this happens in a crypt that has an upward shift in the sweet spot and an expansion of the region below the sweet spot where low APC and high WNT levels occur. In this view, a change in APC and WNT gradients expands the region where fissioning can be initiated so it can occur anywhere along the crypt column including toward the crypt top such that fissioning occurs asymmetrically. But, in *APC* mutant crypts, not only is the symmetry of fissioning abnormal, but the rate of fissioning is increased. Based on our counter-current-like mechanism, changes in APC and WNT gradients due to *APC* mutation lead to an increased WNT gradient (while the APC gradient diminishes). In this view, increased WNT signaling not only increases the rate of crypt fissioning but also causes asymmetric fissioning, which underlies adenoma growth. This view is consonant with studies implicating Wnt/β-catenin signaling in crypt fission because WNT is essential for intestinal SC division (152, 160).

To further understand how our counter-current-like mechanism might relate to aberrant crypt fissioning (asymmetric fissioning and increased rate of fissioning) that drives adenoma development, it is useful to draw parallels between the crypt cycle and the hair follicle cycle. In the hair follicle cycle, hair grows cyclically through three phases: *anagen* is the growth phase; *catagen* the involuting or regressing phase; and *telogen*, the resting or quiescent phase (161). As noted above, there are also three phases in the crypt cycle: the crypt *growth* phase, the crypt *budding/bifurcation* phase, and the crypt *fission* phase. In the hair follicle cycle, WNT signaling maintains the anagen growth phase (162). That WNT signaling and the rate of crypt fission are both increased in *APC* mutant crypts suggests that WNT signaling also has a role in the growth phase of the crypt cycle.

Since, based on our model, “optimal” APC levels in mutant crypts occur higher up the crypt and fissioning occurs asymmetrically at points higher along the crypt axis, APC may also have a role in the budding/bifurcation phase of the crypt cycle. Indeed, it has been proposed that it is APC that normally controls the symmetry of crypt fissioning (60). Our model predicts that at the place where budding first develops at the crypt bottom, cell division is enabled due to localized induction of APC expression that establishes two new gradients which creates a pair of new sweet spots (Figure 2). The enabling of rapid cell division at these new sweet spots creates a motor mechanism that drives growth of the bifurcation upwards toward the crypt top. This proposed mechanism is consonant with biological data showing that increased cell division selectively occurs on both sides of the extending bifurcation in fissioning crypts (163). Moreover, cells staining positively for the Wnt target gene Lgr5 are located at the bottom-most region of the two newly emerging crypts (164). These Lgr5+ cells in bifid crypts appear to be located below both sides of the extending bifurcation. Thus, our model predicts that optimal APC and WNT signaling are crucial to regulating the rate and symmetry of crypt fissioning during the crypt cycle. Therefore, based on our mechanism, changes in APC and WNT that are due to APC mutation alter regulation of the crypt cycle, cause abnormal crypt fission, and drive adenoma development.

## Clinical Significance

Based on our proposed counter-current-like mechanism, it may be possible to develop novel approaches that normalize the APC and WNT gradients, shift the proliferative zone downwards, and thwart the progression of premalignant changes in the *APC* mutant colonic crypt. One can consider targeting APC, but it is unlikely that gene therapy will be efficient enough to transfect wt-*APC* genes into mutant SCs. The alternative is to diminish the WNT gradient. In principle, this can be done by targeting TCF4 or TCF4 target genes such as survivin. Indeed, several agents that inhibit TCF4 activity are already in development (165, 166).

## Conclusion

Our consideration of how *APC* mutations affect the spatial and temporal organization of the colonic crypt led us to propose an APC:WNT counter-current-like mechanism that regulates cell division along the crypt axis. It is a mechanism that explains how *APC* mutations induce proliferative abnormalities that drive colon cancer development. This mechanism also suggests how chemoprevention for this malignancy might be achieved.

## Conflict of Interest Statement

The authors declare that the research was conducted in the absence of any commercial or financial relationships that could be construed as a potential conflict of interest.
